# Mental health impact of the COVID-19 pandemic on the front-line health care workers in a tertiary care hospital in rawalpindi, pakistan

**DOI:** 10.1192/j.eurpsy.2021.699

**Published:** 2021-08-13

**Authors:** U. Zubair, S. Mansoor, T. Mansoor

**Affiliations:** 1 Oak, phoenix care center, Dublin, Ireland; 2 Gender Studies, NUMS, islamabad, Pakistan

**Keywords:** Covid 19, health care workers, mental health

## Abstract

**Introduction:**

In these unprecedented times, the medical workers particularly at the frontlines of the pandemic are vulnerable to psychological trauma rooted in the new demands of their work, as well as the prevailing sense of insecurity and safety concerns for themselves and their loved ones.

**Objectives:**

To explore the mental health impact of the covid-19 pandemic on the front-line health care workers in a tertiary care hospital in Rawalpindi, Pakistan

**Methods:**

Frontline healthcare workers will include all doctors, nurses and paramedical staff performing duties that involve dealing directly with known or suspected cases of COVID-19 at triage clinics, ICUs and isolation wards. Depression Anxiety and Stress Scale (DASS) and Impact of events scale-revised (IES-R) were administered to them to assess the mental health of these frontlines.

**Results:**

A total of 94 frontlines were included in the study. 76(80.9%) had no significant depression while 18 (19.1%) had presence of significant depression. 62 (66%) had no significant anxiety while 32 (34%) had significant anxiety. Female gender and degree of contact with high clinical suspicion cases were significantly related with presence of anxiety and depression among the study participants. Being female, single, a nurse or a paramedic and degree of contact with high clinical suspicion cases was significantly related with presence of stress among the frontlines. People working in ICU or corona isolation unit had more chances of having trauma as compared to other frontlines.

**Conclusions:**

Depression, anxiety, stress and trauma were common among the front liners fighting in this pandemic

